# Combined Autologous Chondrocyte and Bone Marrow Mesenchymal Stromal Cell Implantation in the Knee: An 8-year Follow Up of Two First-In-Man Cases

**DOI:** 10.1177/0963689719845328

**Published:** 2019-05-08

**Authors:** Jingsong Wang, Karina T. Wright, Jade Perry, Bernhard Tins, Timothy Hopkins, Charlotte Hulme, Helen S. McCarthy, Ashley Brown, James B. Richardson

**Affiliations:** 1 Institute of Science and Technology in Medicine (ISTM), Keele University, Staffordshire, UK; 2 Robert Jones & Agnes Hunt Orthopaedic Hospital, Oswestry, Shropshire, UK; 3 Dalian Medical University, Dalian, China

**Keywords:** Knee, cartilage repair, autologous chondrocyte implantation, autologous bone marrow-derived stromal cells

## Abstract

Autologous chondrocyte implantation (ACI) has been used to treat cartilage defects for >20 years, with promising clinical outcomes. Here, we report two first-in-man cases (patient A and B) treated with combined autologous chondrocyte and bone marrow mesenchymal stromal cell implantation (CACAMI), with 8-year follow up. Two patients with International Cartilage Repair Society (ICRS) grade III–IV cartilage lesions underwent a co-implantation of autologous chondrocytes and bone marrow-derived mesenchymal stromal cells (BM-MSCs) between February 2008 and October 2009. In brief, chondrocytes and BM-MSCs were separately isolated and culture-expanded in a good manufacturing practice laboratory for a period of 2–4 weeks. Cells were then implanted in combination into cartilage defects and patients were clinically evaluated preoperatively and postoperatively, using the self-reported Lysholm knee score and magnetic resonance imaging (MRI). Postoperative Lysholm scores were compared with the Oswestry risk of knee arthroplasty (ORKA) scores. Patient A also had a second-look arthroscopy, at which time a biopsy of the repair site was taken. Both patients demonstrated a significant long-term improvement in knee function, with postoperative Lysholm scores being consistently higher than ORKA predictions. The most recent Lysholm scores, 8 years after surgery were 100/100 (Patient A) and 88/100 (Patient B), where 100 represents a fully functioning knee joint. Bone marrow lesion (BML) volume was shown to decrease on postoperative MRIs in both patients. Cartilage defect area increased in patient A, but declined initially for patient B, slightly increasing again 2 years after treatment. The repair site biopsy taken from patient A at 14 months postoperatively, demonstrated a thin layer of fibrocartilage covering the treated defect site. The use of a combination of cultured autologous chondrocytes and BM-MSCs appears to confer long-term benefit in this two-patient case study. Improvements in knee function perhaps relate to the observed reduction in the size of the BML.

## Introduction

Autologous chondrocyte implantation (ACI) is a cell-based therapeutic strategy which has been used for more than 20 years for the treatment of cartilage defects^1^. ACI has shown promising clinical outcomes in terms of pain relief and in delaying joint degeneration^2,3^. ACI has also recently been approved by the National Institute for Health and Care Excellence (NICE TA 477). This historic decision is anticipated to have a profound impact, as ACI is now considered to be the ‘gold standard’ to treat chondral defects (greater than 2 cm in diameter and not previously treated with microfracture). However, there are still some unsolved challenges, such as possible donor-site morbidity^4,5^, and variable structural cartilage regeneration as noted using imaging and histological analyses^6^.

Bone marrow-derived mesenchymal stromal cells (BM-MSCs) have chondrogenic potential which can be enhanced by co-culture with chondrocytes^7,8^. Orozco et al. have confirmed the safety and feasibility of using autologous BM-MSCs to treat cartilage defects^9^. They reported 65% to 78% improvement in knee pain and knee function after implantation, demonstrating improved outcomes compared with conventional treatment (e.g. acupuncture, placebo, debridement, lavage), at 12 months postoperatively, with maintained improvement up to 2 years^10^.

BM-MSCs were initially proposed to maintain their multipotent capacity and contribute directly to repair cartilage formation and engraftment, with MSCs persisting in multiple tissues for as long as 13 months after transplantation^11^. However, another study showed that BM-MSCs migrate to the site of damaged cartilage and are stimulated by local inflammatory cytokines or hypoxia to produce large quantities of growth factors which promote tissue regeneration^12^. Additionally, MSCs have been found to enhance tissue repair by secreting soluble factors which suppress the inflammatory response and stimulate endogenous stem cell proliferation and differentiation^13^.

Combining cultured autologous chondrocytes and BM-MSCs may be of benefit for cartilage regeneration but to our knowledge this has not been reported on in a clinical study. In this first-in-man study we report two cases of such treatment, hereafter referred to as combined autologous chondrocyte and MSC implantation (CACAMI), with 8 years of clinical outcome data.

## Materials and Methods

### Patient Information

Two male patients are described in the study: Patient A (70 years of age, body mass index (BMI) 25.25) and Patient B (65 years of age, BMI 22.09) both presented with unilateral knee osteoarthritis (OA) and full thickness cartilage loss on their medial femoral condyles (International Cartilage Repair Society (ICRS) grade III–IV). Patient A, a keen runner, started to feel right knee pain at 61 years of age and was unable to run at all 9 years later, at the time of treatment. His baseline X-ray showed the complete loss of the medial joint space with sclerosis present in the subchondral bone of the tibia and femur. Patient B began to feel pain in his right knee, without any proximate cause, at the age of 62. At 3 years later, at the time of treatment, he was unable to walk more than a quarter of a mile and could not mobilize around a golf course without the use of a buggy. Both patients were offered knee replacement surgeries but, as keen sportsmen, each wished to avoid arthroplasty, hence they were offered CACAMI. Patient demographic and treatment information, which is distinct for each case, is summarized in Table 1.

**Table 1.** Patient Demographic and Treatment Information.

Case	Defects (number and location)	Previous procedures	Preoperative Lysholm score	Operative notes (at cell implantation)
A	1 Patella 1 MTP 1 MFC	None	50	4 weeks after tissue harvest, intra-articular injection of chondrocytes and BM-MSCs with hyaluronan.
B	1 Patella 1 MTP 1 MFC	Debridement 12 months previously (absent meniscus noted)	75	2 weeks after tissue harvest, traditional ACI with a BM-MSC-seeded Actifit® meniscal transplant.

ACI: autologous chondrocyte implantation; BM-MSC: bone marrow-derived mesenchymal stromal cells; MFC: medial femoral condyle; MTP: medial tibial plateau.

Both patients were fully informed that although cultured chondrocytes and BM-MSCs had each been used individually for many years to treat chondral defects, the combination had not yet been used clinically. The process was explained in full and the patients provided informed consent. Each patient consented to an ethically approved project (REACT 09/H1203/90, approved by South Staffordshire Local Research Ethics Committee, UK).

### Surgical Procedures and Cell Therapy Delivery

The two-stage procedure was performed in both cases by the same surgeon. The first stage was a diagnostic arthroscopy, at which time macroscopically healthy cartilage was harvested from a low load-bearing area of the femoral trochlea in both patients. Additionally, 20 ml of bone marrow was aspirated from the iliac crest.

Isolated chondrocytes and BM-MSCs were expanded in monolayer culture as described previously^14,15^. Briefly, the cartilage biopsy was dissected into 2 mm^3^ pieces and digested in collagenase type II (245 IU/mg dry weight, Worthington, USA) for 16 hours at 37ºC. Chondrocytes were plated out in Dulbecco’s modified Eagle’s medium (DMEM)/F12 supplement with 20% autologous serum, 50 µg/ml ascorbic acid (Sigma Aldrich, Poole, UK) and antibiotics at a seeding density of 5 × 10^3^ cells/cm^2^. Cells were passaged at 70% confluence and reseeded in culture medium containing 10% autologous serum. Then chondrocytes were passaged a further 1 or 2 times and prepared for implantation within 4 weeks. To culture BM-MSCs, mononuclear cells were isolated by density gradient centrifuge (Lymphoprep^TM^, Fresenius Kabi Norge AS, Norway) were seeded at a density of 20 million cells per 75 cm^2^ flask in DMEM/F12 supplement with 15% autologous serum and antibiotics. BM-MSCs were passaged at 70% confluence and cultured in medium with 10% autologous serum. The cells were subcultured for a further 1–2 passages and implanted within 4 weeks. Cartilage and bone marrow aspiration harvest and final cell yields are summarized in Table 2. Chondrocytes and BM-MSCs were released for implantation upon conformance to expect morphological characteristics and if cell populations were at least 90% viable, as assessed by trypan blue exclusion (as described in detail in a published current clinical trial)^16^.

**Table 2.** Bone Marrow and Cartilage Cell Isolation and Growth Kinetic Information.

Case	Bone marrow			Cartilage			Culture time (days)
	*Volume* *(ml)*	*Cell number*		*Weight* *(mg)*	*Cell number*		
		*Initial MNC*	*Implanted BM-MSCs*		*Initial*	*Implanted*	
A	20	4×10^6^	8 ×10^6^	301	4.9×10^5^	4×10^6^	28
B	20	3×10^6^	6 ×10^6^	286	4.0×10^5^	4×10^6^	14

BM-MSC: bone marrow-derived mesenchymal stromal cell; MNC: mononuclear cell.

**Fig. 1.**
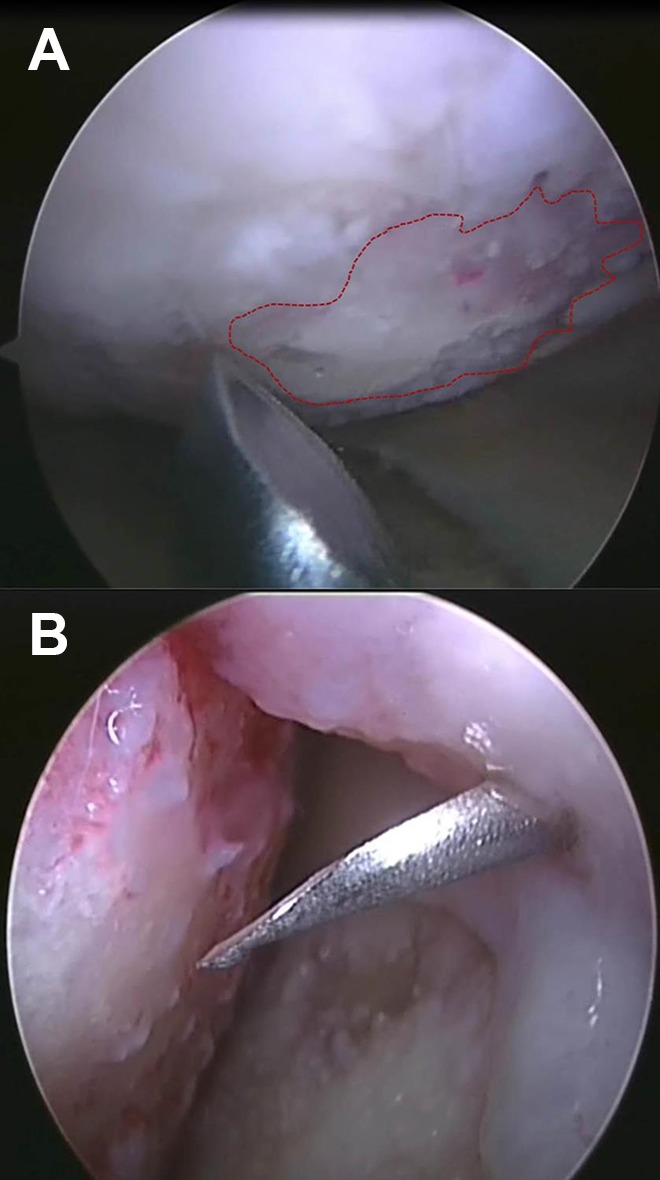
Arthroscopic images of the medial femoral condyle in Patient A. (A) A full-thickness cartilage defect was observed at the time of cell implantation (red dashed-line). (B) Following the debridement of the lesion, the cultured chondrocytes and MSCs were injected into the synovial cavity, in combination with hyaluronan. MSC: mesenchymal stromal cell.

Patient A underwent a second arthroscopic procedure, in which the chondral defect on the medial femoral condyle was debrided. After this, combined autologous chondrocytes and BM-MSCs were injected into the joint, followed by hyaluronic acid (60 mg in 3 ml Durolane; Fig. 1). Patient B’s second stage surgery involved a parapatellar arthrotomy, in which the defect edges were cut vertically using surgical blades. A porcine collagen type I/III membrane (Chondrogide, Geistlich Biomaterials, Wolhusen, Switzerland) was sutured to the surrounding healthy cartilage using 6.0 Vicryl (Ethicon Leeds, UK). The edges of the patch were sealed to the edges of the defect with fibrin glue (Tisseel, ImmunoAG, Vienna, Austria). Finally, a suspension of cultured chondrocytes was injected underneath the patch, after which a BM-MSC-seeded medial Actifit^®^ polyurethane meniscal scaffold (Orteq Sports Medicine, London, UK) was transplanted.

### Clinical Assessments

**Fig. 2.**
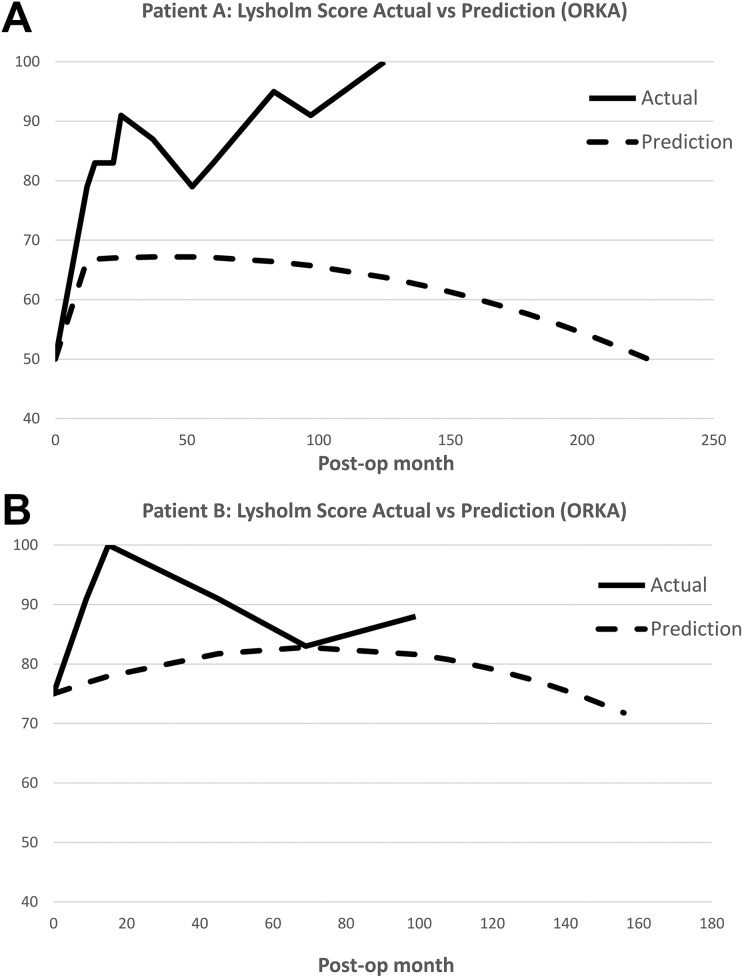
A comparison of the longitudinal, postoperative actual Lysholm score and ORKA prediction score. (A) For Patient A, the actual score gradually increased to 100, surpassing predicted scores at each time point. (B) For Patient B, the actual score increased initially to 100, after which the score decreased to 83, matching ORKA predictions. Subsequently the actual score then increased again to 88 at the 5-year time point. ORKA: Oswestry risk of knee arthroplasty.

**Fig. 3.**
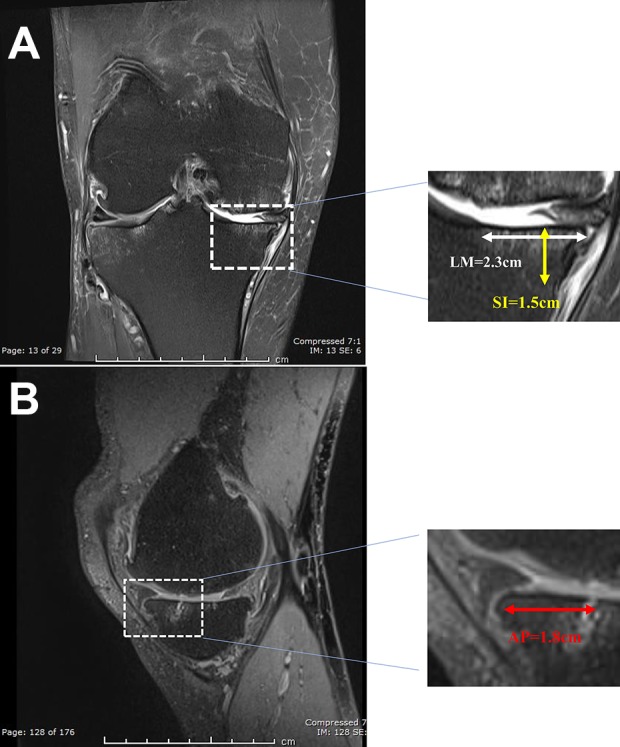
Measurement of the bone marrow lesion volume on MRI within the medial tibial plateau of patient A. (A) In the coronal image, the LM dimension (2.3 cm) and SI dimension (1.5 cm) of the lesion are measured. (B) In the sagittal image the AP dimension of the same lesion (1.8 cm) is measured. AP: anterior-posterior; LM: lateral-medial; MRI: magnetic resonance imaging; SI: superior-inferior.

Each patient was evaluated preoperatively and postoperatively up to 8 years, using a modified Lysholm score^17^. Actual postoperative scores were compared with scores predicted using the Oswestry risk of knee arthroplasty (ORKA) score. The ORKA score^2^ is a web-based application that uses baseline information about the patient’s age and sex, location of and the number of cartilage defects and baseline knee function score to predict a patient’s risk of needing knee arthroplasty (Fig. 2). Postoperative magnetic resonance imaging (MRI) scans at 2, 6, 13, 39, 49, 60 and 86 months for Patient A and at 5, 17, 25 and 52 months for Patient B were undertaken to assess the cartilage and bone using two scoring systems. The magnetic resonance observation of cartilage repair tissue (MOCART) score, which evaluates cartilage repair tissue^18^ and the whole-organ magnetic resonance imaging score (WORMS). WORMS provides whole-organ evaluation of the total knee joint by assessing not only the cartilage, but also various other structures, including the menisci, ligaments, subchondral bone and bone marrow^19^. For each patient, longitudinal postoperative MOCART and WORMS scores were evaluated by a radiologist with significant experience in OA and ACI (Table 3). A higher MOCART score indicates better cartilage repair, whereas a lower WORMS value indicates a better preserved joint. Bone marrow lesions (BMLs) were also assessed and measured as described previously^20^ and depicted in Fig. 3.

**Table 3.** A Summary of the MRI Scores (WORMS and MOCART) for Patient A and B.

Patient	Months Post-op	WORMS (0–332)	MOCART (0–100)
A	3	133.5	10
	14	112	15
	61	124	15
B	5	138	75
	17	138	60
	52	143.5	60

MOCART: magnetic resonance observation of cartilage repair tissue; MRI: magnetic resonance imaging; WORMS: whole-organ magnetic resonance imaging score.

A second-look arthroscopy was performed in Patient A, 14 months after cell therapy, in which a biopsy of repair cartilage was taken. The sample was snap-frozen in liquid nitrogen-cooled hexane and sectioned at 7 µm thickness onto poly-L-lysine coated glass slides. Sections were stained with hematoxylin and eosin or toluidine blue to assess general morphology and proteoglycan content, respectively. Collagen fiber orientation was examined under polarized light to differentiate between hyaline cartilage and fibrocartilage.

## Results

Both patients reported improvements, as measured by pain reduction, within a year of treatment. No complications from surgery were noted in either patient. Further improvements continued up until the time of reporting this study (8-year follow up) and both patients currently remain physically active without joint replacement, with Lysholm scores of 100 and 88, for patients A and B respectively (Fig. 2). Patient A returned to running competitively and has recently taken up swimming. Patient B continues to compete in cycling races. His main residual symptom at final review is pain that develops after driving for more than 1 hour.

**Fig. 4.**
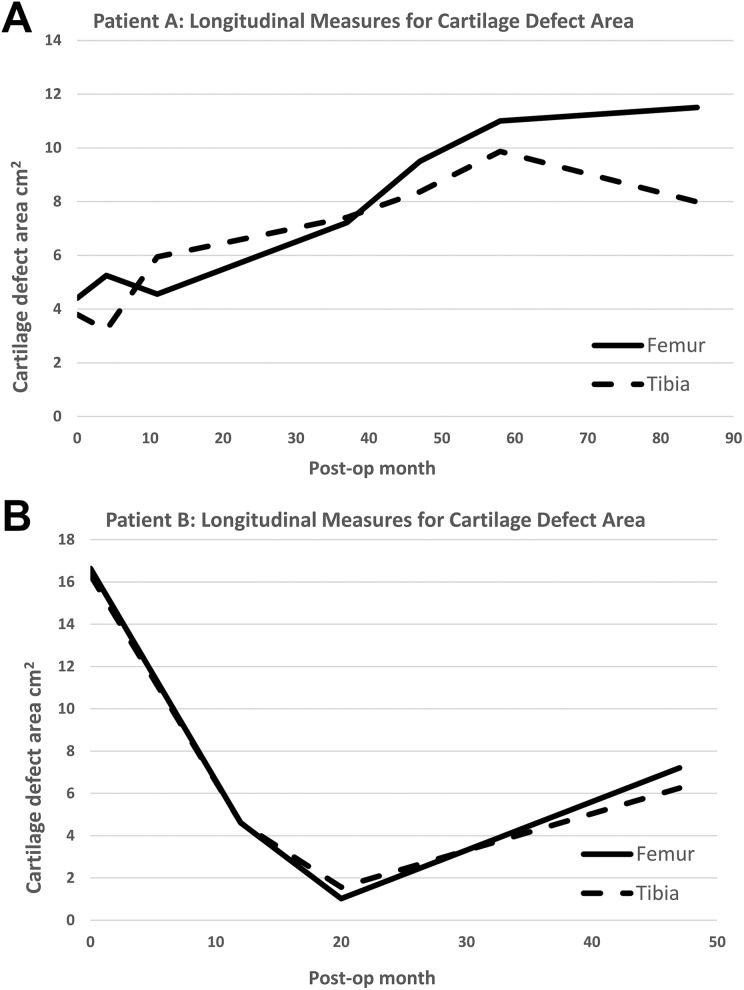
Cartilage defect size measured from MRIs for Patients A and B. (A) The area of the cartilage defects gradually increased over time for Patient A, whereas the size of cartilage defects decreased for the first 2 years, but increased again thereafter for Patient B (B). MRI: magnetic resonance imaging.

**Fig. 5.**
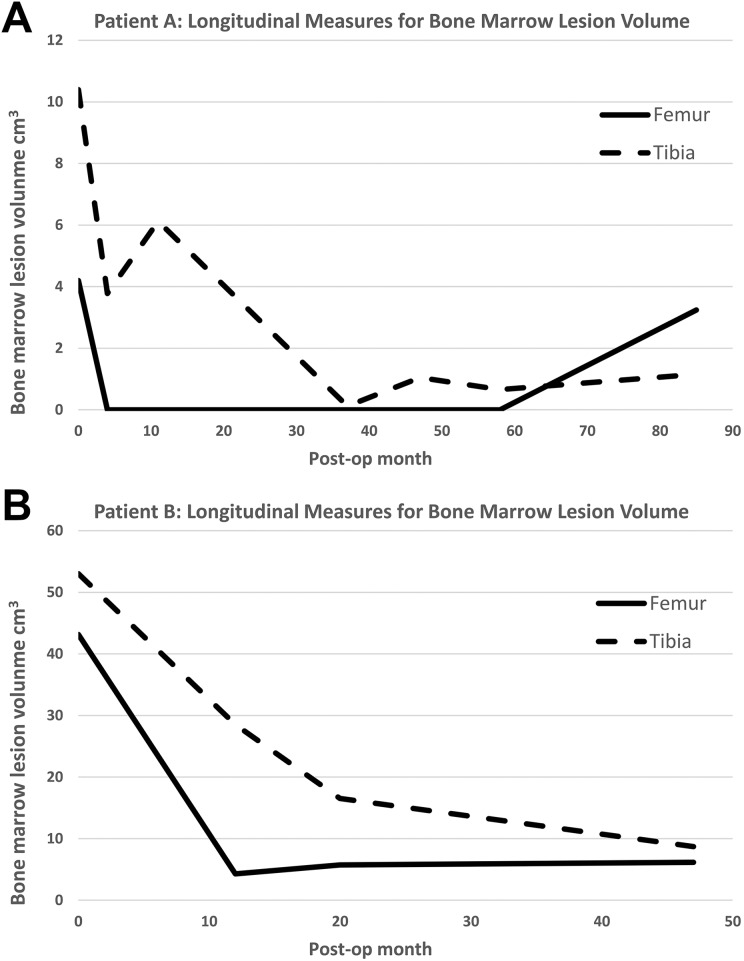
BML size measured from MRIs for Patients A and B. (A) The BMLs continuously decreased in volume, particularly on the tibial side for Patient A, whereas the BMLs continuously decreased in volume both on the tibial and femoral sides for Patient B (B). BML: bone marrow lesion; MRI: magnetic resonance imaging.

Patient A showed progressive enlargement of chondral defects identified on MRI (Fig. 4A), whereas in Patient B the defect area declined initially and increased slightly 2 years after implantation (Fig. 4B). In both patients, the size of BMLs decreased gradually over time in the tibia as well as the femur. This was especially evident in Patient B, in whom more than 80% of the BMLs disappeared (Fig. 5A and B).

MOCART and WORMS scores at three different times are shown in Table 3. For patient A, the WORMS score fluctuated over time, while the MOCART only marginally improved from 10 to 15. However, for patient B both scores consistently deteriorated over time, but MOCART scores (from 75 to 50) were consistently higher than for Patient A. Additionally, there was no evidence demonstrating the presence of the scaffold or new meniscal tissue in postoperative MRI at 5-month follow up and beyond for patient B.

At second-look arthroscopy for Patient A, 12 months after surgery, the treated defect on the medial femoral condyle was observed to be generally well healed with firm repair tissue on probing, but with a slightly irregular surface. In contrast, the tibial plateau repair cartilage appeared soft but smooth. Histologically, a thin layer of fibrocartilage had formed in the treated defect on the medial femoral condyle, measuring 0.5 mm in depth where previously the bone had been exposed.

## Discussion

Our previous case report showed the feasibility of combining ACI with bone graft to treat a major osteochondral defect in the hip following trauma^21^. Combining cultured autologous chondrocytes and BM-MSCs may be of benefit for cartilage regeneration but to our knowledge has not been reported previously in a clinical study. We believe this is the first case report to demonstrate the safety of using combined autologous chondrocytes and autologous BM-MSCs to treat cartilage defects. Clinical symptoms improved dramatically in both patients treated, up to 8 years follow up. We have previously identified six important predictive factors (age, sex, defect number and location, previous surgery, preoperative Lysholm score) in ACI-treated patients, which appear to influence progression to arthroplasty. The combination of these six parameters was used to construct an ORKA index to predict the survival of ACI repair^2^. In this study, both patients were over 60 years old and had multiple defects including patella sites, as well as low preoperative Lysholm scores, which when using the ORKA assessment indicates a poor prognosis. However, both patients’ postoperative Lysholm scores were consistently higher than ORKA predictions.

The patient-reported Lysholm score may have some limitations associated with its subjectivity. However, our group and many others consistently use such a subjective patient-reported outcome as the primary outcome of clinical trials^16,17,22^. The reason being that ultimately, how the patient feels about their knee function, is the most important clinical observation and metric of treatment success/failure. Moreover, several studies have shown that there is no correlation between subjective functional outcome such as Lysholm score and objective measures such as histological analyses^23,24^. We have taken measures to help to reduce the limitations associated with the use of subjective scores, including for comparison objective measures (clinical imaging scores) and where possible, histological observations. In addition, we have not relied upon a single Lysholm outcome, but include several, longitudinal postoperative scores for each patient which helps to add further confidence.

The second-look arthroscopy and histological assessments showed that the cartilage defects in Patient A were repaired with a thin layer of fibrocartilage which was not noted at 1-year follow-up MRI. Patient B’s cartilage defect sizes were reduced in the early postoperative assessments in contrast to tissue to Patient A, in which the defect sizes increased progressively after treatment. This difference could be attributed to the use of a patch covering the treatment site in Patient A, or to the difference in cell delivery method. Patient B’s injected BM-MSCs may have migrated out of the implanted Actifit^®^ scaffold and exerted their effects elsewhere within the joint. However, no firm conclusions regarding which cell delivery method represents the better cartilage repair technique can be drawn.

The size of the BMLs in both patients gradually decreased over time. BML is a technical term to describe the high-signal intensity pathological change on MRI^25^. There are multiple studies demonstrating the positive correlations between BMLs and the pathogenesis of knee pain^26–28^. A large prospective cohort study of 358 patients in Korea showed the prevalence of BMLs in patients with knee OA was 80.3%. After adjusting for age, sex and BMI, the presence and severity of BMLs in the medial compartment were significantly associated with knee pain^27^. Zhang et al. reported similar results, showing that the changing size of BMLs was related to knee pain fluctuations, and the reduction of BMLs could lower the risk of frequent knee pain^29^. Although Sower et al. demonstrated a poor relationship between the presence of knee pain and the presence of BMLs on MRI^30^, a systematic review on 22 studies showed moderate evidence that BMLs were associated with knee OA pain^31^.

In a recent systematic review, the clinical outcome of 347 patients who had undergone Actifit^®^ (meniscal) implantation without cells was evaluated and graft failure occurred in 9.9% of patients at a mean follow up of 40 months^32^. For Patient B, treated with CACAMI and Actifit^®^, postoperative MRI at 5-month follow up demonstrated no obvious signal for the presence of the scaffold or new meniscal tissue, indicating possible resorption of the scaffold. In comparison, Gelber et al. reported that patients with no chondral injuries showed an improved size and morphology of transplanted Actifit^®^ scaffolds using MRI at a median follow up of 39 months (range 25–63)^33^.

In conclusion, we believe these two case studies suggest that combination of cultured autologous chondrocytes and BM-MSCs appears to confer improved symptoms at least in these two patients. However, despite the improvement in clinical outcome scores, physical joint failure as assessed by MRI is progressing in both patients. Whether this might have progressed with advancing age at a greater rate had the cells not been implanted, will only be resolved in a full clinical trial. In summary, these findings support the hypothesis that autologous BM-MSCs stimulate a beneficial host response, reducing pain, perhaps by influencing the subchondral bone and reducing the size of BMLs.
